# Insights into Microbiota–Host Crosstalk in the Intestinal Diseases Mediated by Extracellular Vesicles and Their Encapsulated MicroRNAs

**DOI:** 10.3390/ijms252313001

**Published:** 2024-12-03

**Authors:** Yan Zeng, Yulong Yin, Xihong Zhou

**Affiliations:** 1 Key Laboratory of Agro-Ecological Processes in Subtropical Region, Institute of Subtropical Agriculture, The Chinese Academy of Sciences, Changsha 410125, China; zengyan23@mails.ucas.ac.cn; 2 College of Advanced Agricultural Sciences, University of Chinese Academy of Sciences, Beijing 100049, China

**Keywords:** extracellular vesicles, inflammation, microbiota, microRNAs

## Abstract

Microorganisms that colonize the intestine communicate with the host in various ways and affect gut function and health. Extracellular vesicles (EVs), especially their encapsulated microRNAs (miRNAs), participate in the complex and precise regulation of microbiota–host interactions in the gut. These roles make miRNAs critically important for the prevention, diagnosis, and treatment of intestinal diseases. Here, we review the current knowledge on how different sources of EVs and miRNAs, including those from diets, gut microbes, and hosts, maintain gut microbial homeostasis and improve the intestinal barrier and immune function. We further highlight the roles of EVs and miRNAs in intestinal diseases, including diarrhea, inflammatory bowel disease, and colorectal cancer, thus providing a perspective for the application of EVs and miRNAs in these diseases.

## 1. Introduction

The intestine is colonized by thousands of microorganisms, and healthy gut microbial communities are beneficial for intestinal function and necessary to resist the invasion of external pathogens and harmful substances. Microbiota–host crosstalk refers to disease resistance, immune system homeostasis, and intestinal maturation [1]. However, the key regulatory mechanism of the intestine–microbiota crosstalk remains to be elucidated. Owing to advances in sequencing technology, multiple biogenic pathways and small RNA activities have been discovered over the past decades. Small RNAs, including microRNAs (miRNAs), siRNAs, and PIWI-associated RNAs, have been widely studied [2], with miRNAs being the most studied. MiRNAs are endogenous, non-coding, single-stranded RNAs derived from a conserved short hairpin structure consisting of approximately 22 base pairs. MiRNAs recognize post-transcriptional genes through base complementary pairing and guide genes to degradation or silencing [3]. Extracellular vesicles (EVs) are extracellular membrane-bound vesicles with diameters ranging from 40 to 100 nm that are widely present in various biological fluids. Different types of molecules can be present in EVs, such as proteins, lipids, and nucleic acids [4]. The prediction and early diagnosis of gastrointestinal diseases remain significant challenges in contemporary research. It is crucial to identify an efficient and practical approach to address these issues. Recently, numerous studies have focused on the roles of EVs and encapsulated miRNAs, suggesting their potential in advancing this field. They alter the composition of the gut microbiota and mediate the occurrence of intestinal diseases. EVs and miRNAs participate in the gut microbiota crosstalk and bind to key factors in important pathways, thereby regulating gene expression [5]. Moreover, the synergistic use of miRNAs in conjunction with traditional biomarkers has shown a significant increase in sensitivity compared to the use of individual biomarkers alone [6]. As a result, EVs and encapsulated miRNAs are increasingly being employed as biomarkers for disease prediction, diagnosis, and treatment.

Accordingly, this manuscript summarizes the underlying mechanisms of EVs and their encapsulated miRNAs in mediating gut–microbiota crosstalk, as well as their roles in intestinal diseases, providing a basis for using miRNAs to regulate host intestinal health.

## 2. Crosstalk Between Microbiota–Host in the Intestine

Recently, the role of the microbiota in mammalian intestinal health has received increasing attention. Many studies have proven that a microbiome is presented in the gut prior to birth, and many factors affect intestinal microbiota as the host matures [7,8]. The large intestine is the major site of microbial fermentation, whereas the small intestine contains microbiota related to immune functions [9,10]. The dynamic balance of the microbiota plays a substantial role in resisting intestinal diseases, such as diarrhea and inflammatory bowel diseases (IBD) [11]. Therefore, understanding how microbiota interacts with intestinal cells is beneficial for the prevention and treatment of diseases.

In general, crosstalk between the microbiota and intestine is mediated by microbial metabolites. Bacteriocin secreted by *Lactobacillus* can resist diarrhea by enhancing intestinal fluid absorption and decreasing intestinal fluid secretion [11], while the pathogenic effects of *E. coli* can be mitigated by bacteriocins secreted by commensal probiotic *E. coli* strains of human origin, both in vitro and in vivo [12]. The widely researched microbial metabolites, short-chain fatty acids (SCFAs), can maintain intestine barrier function by increasing the abundance of occludin and claudin-1 in the duodenum and ileum and decreasing the protein abundance of IL-1β in the colon, thus improving intestinal immune function [13]. Many receptors located on intestinal cells react with gut microbes and their metabolites. Lipopolysaccharides (LPS) from Gram-negative bacteria are recognized by toll-like receptor 4 (TLR4) and cluster of differentiation 14 (CD14) in epithelial cells, inducing the secretion of proinflammatory cytokines [14,15]. Peptidoglycan, found in microbial cell walls, can bind to peptidoglycan recognition proteins (PGLYRP1–PGLYRP4), which are expressed mainly in intestinal immune cells and epithelial cells, to exert antibacterial activity [16]. Bacterial adhesins, such as fimbriae F4 and F18, expressed in enterotoxigenic *E. coli* can bind to various receptors on epithelial cells, further activating the immune response and mediating crosstalk between microorganisms and the intestine [17].

Quorum sensing (QS) is a population density-dependent physiological behavior that regulates gene expression. Typically, when the concentration of the autoinducer released by bacteria reaches a certain threshold, it alters the expression of specific genes in bacteria [18]. The main signaling molecules adopted by different bacterial species are N-acyl-homoserine lactones, autoinducing peptides, and autoinducer 2 (AI-2) [19]. Quorum sensing is involved in a variety of biological processes and affects the spatial distribution of the intestinal flora [20]. Recently, numerous studies in quorum-sensing have been conducted. Pathogenic bacteria, such as *E. coli*, induce the death of intestinal epithelial cells by secreting AI-2 [21], and the activity of AI-2 decreases in the presence of *Lactobacillus acidophilus* strain 30SC cell extract [22]. The inhibition of AI-2 molecules also reduces the ability of lactobacilli to adhere to the host gut [23].

**Figure 1:**
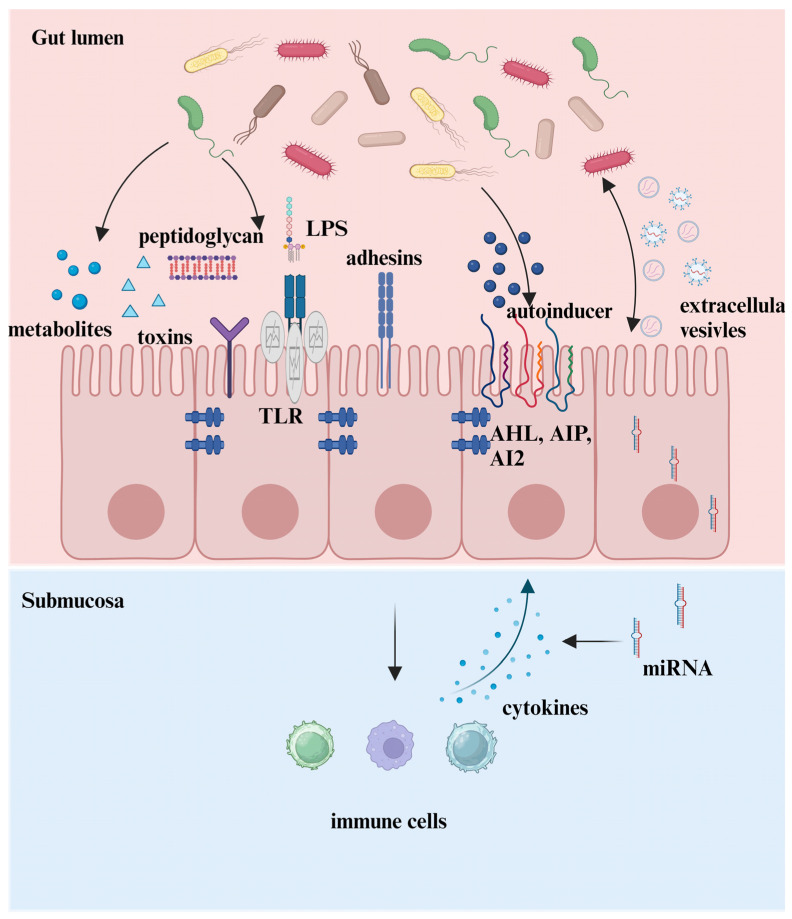
The crosstalk between microbe–host in the intestine. Microbiotas interact with the host through five manners. (1) Microbiota-sourced metabolites. (2) Components of microbiota bind to receptors on epithelial cells. (3) Microbiota colonizes in the host by bacterial adhesins. (4) Microbiotas interact with the host via quorum sensing. (5) The microbiota and host interact bilaterally via EVs and their encapsulated miRNAs.

In addition to these mediators, which have been extensively studied and reviewed [24], the functions of EVs and their encapsulated miRNAs have become a new focus as advances in sequencing technology (Figure 1). An increasing number of publications have greatly expanded our understanding of their roles in the microbe–intestine crosstalk in recent years.

## 3. Extracellular Vesicles Mediate Microbiota–Intestine Crosstalk

EVs produced in the endosomes of eukaryotic cells can be classified based on the vesicular budding pathway from the cell plasma membrane. One is directly secreted from the cell membrane, defined as microvesicles and apoptotic bodies, and the other is exosomes, which primitively bud inward from the cell plasma membrane and form endosomes. These endosomes invaginate and form many small vesicles called multivesicular bodies (MVBs). Ultimately, MVBs fuse with the plasma membrane and release its intraluminal endosomal vesicles [4,25,26]. EVs are equipped with heterogeneous macromolecules such as proteins, nucleic acids, and lipids. Among these cargos, mRNAs and miRNAs carried by EVs play critical roles in cell–cell communication and have attracted the most attention [25]. As reported previously, the opportunities for different miRNAs to enter EVs vary. For example, miR-150 and miR-142-3p preferentially enter EVs [27]. EVs expression varies under different conditions. Four major underlying mechanisms for the sorting of miRNA into EVs have been reported [4]: (1) the neural sphingomyelinase 2-dependent pathway; (2) the miRNA-induced silencing complex-related pathway; (3) the 3′-end of the miRNA sequence-dependent pathway; and (4) the miRNA motif and sumoylated heterogeneous nuclear ribonucleoprotein-dependent pathway. These mechanisms emphasize the importance of specific exosomal miRNA sequences that can guide miRNAs into EVs. Usually, miRNAs encapsulated in EVs incorporate with their target genes in recipient cells to regulate their function [28]. Additionally, utilizing in vivo and in vitro models, tumor-secreted EVs, miR-21 and miR-29a, were found to act as glands binding to TLR, indicating that exosomal miRNAs can exert their functions as paracrine agonists [29].

The crosstalk between the intestine and microbiota relies heavily on the secretion and transportation of EVs. EVs are crucial carriers of bioactive molecules from the microbiota, capable of crossing the intestinal barrier to interact with host cells [1]. EVs have three fates after formation. Surface transmembrane proteins on EVs directly bind to receptors on target cells [30]. Second, the transported cargo is released into the target cells through endocytosis. The specific mechanisms are clathrin-dependent, caveolae-dependent, phagocytosis, macropinocytosis, and lipid raft-mediated uptake [31]. Third, the EVs develop into endosomes via endocytosis, mature into lysosomes, and undergo degradation [4].

Diet-derived EVs have a profound influence on the composition of the host intestinal microbes. Ginger-derived EVs increase the abundance of *Lactobacillaceae* and *Bacteroidales* and decrease the abundance of *Clostridiaceae* in the intestine; IL-22 is induced by ginger-derived EVs to improve intestinal barrier function and ameliorate colitis [32]. These results suggest that externally sourced EVs help maintain healthy gut function by improving microbiota–host cell interactions. Host cells can secrete EVs, which are considered the connecting kingdom for host and gut microbiota intercommunication. Notably, EVs secreted by host cells can deliver miRNAs to bacteria and bind to bacterial genes, affecting their abundance and phenotype [33]. Intestinal epithelial cells (IECs) secrete EVs with high levels of miR-130a and miR-30c expression to inhibit autophagy-related proteins, including ATG5 and ATG16L1, thereby favoring *E. coli* intracellular replication and colonization [34]. Circular RNAs of HIF1α encapsulated in EVs interact with the KH domain of IGF2BP3 in a m6A-modified manner, inhibiting the cell cycle and consequently suppressing pathological colonization in the intestine [35]. Importantly, the miRNAs contained in the IEC-derived EVs correlated with gut microbes. For instance, EVs containing miR-200b-3p, miR-200b-5p, and miR-26a-5p show a negative correlation with *Dubosiella* and *Lactobacillus* in the colon of an acute colitis model [36]. Treatment with the *Bacillus amyloliquefaciens SC06* probiotic promotes miR-222 expression in EVs derived from porcine epithelial cell lines (IPEC-J2) and stimulates macrophage polarization to M1 [37].

Bacteria can also secrete bacterial extracellular vesicles (BEVs). BEVs are double-layered vesicles with diameters of 20–300 nm. Although BEVs differ from EVs, they can cross the intestinal barrier and enter cells via endocytosis, macropinocytosis, and endocytosis. Thus, BEVs are essential mediators in the gut microbiota crosstalk by transporting bacterial molecules, including LPS, peptidoglycans, lipids, proteins, nucleic acids, and even toxins, to host intestinal cells [38]. Small RNAs (sRNAs) are the dominant components of BEVs. These molecules play an undeniable role in the microbe–intestine crosstalk [39]. sRNA71 is abundant in *Lactobacillus plantarum*-derived EVs based on small RNA sequencing. It is validated that cellular protein expression is adjusted by sRNA71 by binding to the 3′-untranslated region (UTR) of target mRNAs in vitro, indicating its role in microbe–host interactions [40]. Unlike eukaryotes, BEV-encapsulated sRNA regulates host genes by relying on Hfq instead of the miRNA-induced silencing complex (RISC) [31]. Hfq is a hexameric protein conserved among Gram-negative and -positive bacteria that contains a portal structure, distal structure, lateral rim, and C-terminal domains. Usually, the portal structure binds to single-stranded sRNA, whereas the distal structure binds to mRNA [41]. BEVs are beneficial to intestinal bacterial colonization and proliferation by delivering adhesion factors and polysaccharide-degrading enzymes [42,43]. Additionally, BEVs are closely involved in regulating the host intestinal immune response and have intricate connections with various diseases. The connection between BEVs and immune function was confirmed as forty-eight *Bacteroides thetaiotaomicron* EV proteins were found to interact with host immune cells [44]. Unfortunately, some BEVs can impair the host innate immune response to promote bacterial colonization, such as *Vibrio cholerae*, which EVs can induce miR-146a expression to reduce the innate immune reactions in IEC and inhibit inflammation to facilitate host colonization [45]. However, BEVs derived from Gram-negative *E. coli Nissle 1917* and its commensal, ECOR12, contain peptidoglycans, which can activate the NF-kB pathway in a NOD1-dependent manner in IEC [46]; BEVs derived from *Fusobacterium nucleatum* exhibit multiple outer membrane protein porin FomA, which can activate the NF-kB pathway through a TLR2-dependent manner in IEC [46]. The activation of these two pathways helps the host maintain immune homeostasis and resist invasion by exogenous pathogenic bacteria. Macrophages are key intermediaries in BEV-mediated inflammatory responses, which have a superior ability to maintain the gut barrier integrity [47]. EVs derived from *Lactobacillus johnsonii* modulate macrophage conversion to the M2 phenotype by suppressing the NLR family pyrin domain-containing 3 (NLRP3) signaling pathway in IEC, which promotes gut barrier repair [48]. BEVs derived from *Limosilactobacillus mucosae* profoundly alleviate diarrheal disease symptoms by regulating macrophage phenotypes in germ-free mice [49]. *Bifidobacterium longum* exerts anti-inflammatory effects by activating the immune system [50]. BEVs derived from *Bifidobacterium longum* are rich in anti-inflammatory proteins, including ABC transporters, quorum-sensing proteins, and extracellular solute-binding proteins. Notably, BEVs can induce secretion of the anti-inflammatory cytokine IL-10, which emphasizes their immunomodulatory effects [51]. However, certain pathogenic bacteria deliver toxins to their hosts, resulting in inflammation and proptosis. For instance, BEVs from *Neisseria gonorrhoeae*, uropathogenic *E. coli*, and *Pseudomonas aeruginosa* can activate macrophages to induce mitochondrial apoptosis and NLRP3 inflammasome activation [47]. In addition, amino acids can stimulate microorganisms to produce EVs, thereby alleviating host homeostatic dysfunction. Glycine promotes *Bacteroides acidifaciens* to secrete BEVs and alters their protein profile, considerably enhancing intestinal barrier repairment [52]. The complex interaction between BEVs and the intestine relies on bacteria, intestinal physiological conditions, and other substances, and the use of BEVs to maintain intestinal health and treat diseases is a long and arduous task (Table 1).

## 4. MiRNAs Mediate Microbiota–Intestine Crosstalk

**Figure 2:**
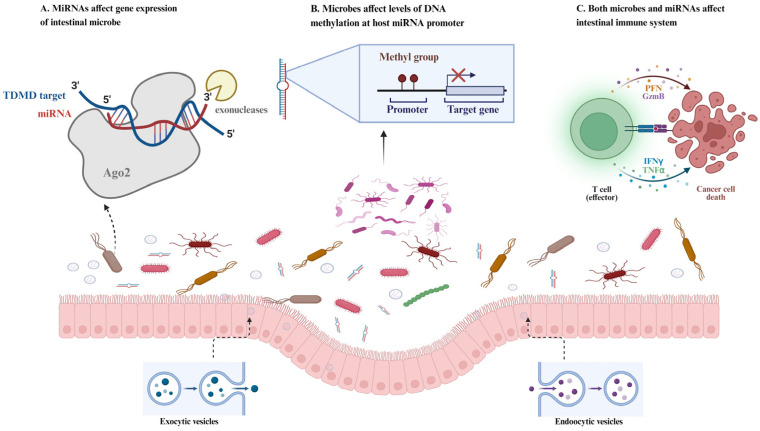
The microbe–host crosstalk mediated by miRNAs in the intestine. (**A**) MiRNAs target key genes in microbiota to regulate their abundance and metabolism. (**B**) Microbes affect the level of DNA methylation at the host miRNA promoter to regulate the expression of miRNAs. (**C**) MiRNAs interact with microbiotas and then affect the intestinal immune system.

Intestinal epithelial and hopx-positive cells secrete various miRNAs and are the main sources of intestinal miRNAs [53]. Canonically, the degree of complementarity between miRNAs and their target mRNA determines their fate. Generally, when there is complete complementarity, the target mRNA is degraded, whereas, if the miRNA is not fully complementary to the target mRNA, translation is hindered [54]. Notably, when miRNAs interact with the gene promoter, 5′-UTRs, and coding sequences, they may also lead to activation of gene expression or other consequences (Figure 2) [55]. MiRNAs can be transported via EVs or present in EV-free forms associated with high-density lipoproteins or argonaute protein [56].

There are strong interactions between miRNAs and the intestinal microbiota. First, miRNAs mediate microbial metabolism. Succinate is an important metabolite of *Prevotella*, and its excessive accumulation in the colon usually causes diarrhea. One study found that ssc-miR-425-5p and ssc-miRNA-423-3p reduce the succinate concentration by targeting the gene of a key enzyme, fumarate reductase, thereby alleviating diarrhea [57]. In addition, miRNAs can directly alter the microbiota abundance to treat inflammation. MiRNA-142a-3p can promote the growth of *Lactobacillus reuteri* to prevent colitis induced by dextran sulfate sodium (DSS), and its potential targets are locus tags LREU_RS06530 and LREU_RS03575, which encode DNA polymerase I and primase, respectively [58]. Host miRNAs enter bacteria and specifically alter bacterial transcripts. When *Fusobacterium nucleatum* is co-cultured with human miR-515-5p, the ratio of *F. nucleatum* 16S rRNA/23S rRNA transcripts increased, whereas mutated miRNAs did not exhibit this effect [53]. Fecal miRNAs, which can influence suitable intestinal flora, are also popular in research. IEC-miRNA-deficient mice exhibit a completely different microbial composition from that of wild-type (WT) mice, and WT fecal miRNA transplantation restores this alteration. Moreover, WT fecal miRNA transplantation can alleviate the symptoms of, and has the potential to treat, colitis [53].

In turn, intestinal microbiota can regulate the expression of miRNAs, thereby altering the intestinal homeostasis. Compared to germ-free mice, specific pathogen-free mice have a more abundant miRNA profile [53]. Probiotic treatment alleviates macroscopic colonic damage in mice by regulating miRNA expression. *Lactobacillus fermentum* increases miR-159 and miR-143 expression, thereby preserving the intestinal barrier function [59]. *Akkermansia muciniphila* colonizing the intestinal mucosa is beneficial for maintaining the intestinal barrier and a healthy mucus layer, and a decrease in its abundance is correlated with the development of diseases such as IBD, appendicitis, and obesity [60]. *A. muciniphila* can upregulate the expression of miR-143 and miR-145, which promote IEC regeneration and barrier integrity [61]. Pathogenic bacteria can alter miRNA expression to improve cell survival, replication, and persistence [62]. *Salmonella enterica* is one of the main pathogenic bacteria that cause gastrointestinal disease, mortality, and substantial economic loss to the livestock industry [63]. After intranasal inoculation with *S. enterica*, 62 differentially expressed miRNAs were identified in vivo in whole blood. The expression of miR-214 decreased, whereas that of miR-331-3p increased. MiR-214 and miR-331-3p induce immune responses against *S. enterica* by targeting important immune sites, including SLC11A1, LILR-like, and VAV2 [63]. MiR-125a is a direct target of the major histocompatibility complex-class I component PSMB8, which is also decreased in mesenteric lymph nodes treated with *S. enterica* [64]. Other pathogenic bacteria, such as *P. aeruginosa* and *Helicobacter pylori*, also influence miRNA expression in the gastro-intestine [65,66]. Several studies have highlighted the potential mechanisms of action. Bacteria can regulate the levels of DNA methylation, a type of epigenetic modification, via the microbial metabolite SCFA [67]. Additionally, miRNAs respond to immune-related factors. For example, commensal bacteria downregulate miR-10a expression in dendritic cells by stimulating TLR ligands in a myeloid differentiation primary response gene 88 (MyD88)-dependent manner [68]. However, the exact mechanism by which intestinal microbiota affects miRNA expression needs to be further elucidated (Table 2).

## 5. MiRNAs and Gut Microbiota Interactions in Intestinal Diseases

**Figure 3:**
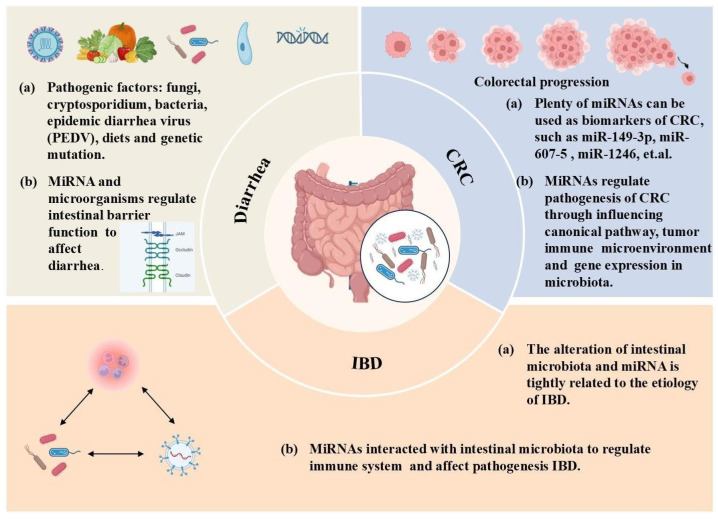
The dominant function of extracellular vesicles and their encapsulated miRNAs in intestinal diseases. Interactions among microbiota, miRNAs, and the immune system involves the pathogenesis of IBD. MiRNAs interact with microbiota to affect intestinal barrier function in diarrhea. MiRNAs influence the canonical pathway, tumor immune environment, and gene expression in microbiota in CRC.

Research has focused on the role of EVs and miRNAs in disease prediction and diagnosis because of their sensitivity to alterations in the homeostasis. Using EVs and miRNAs to mediate intestinal–microbiota crosstalk is expected to provide new avenues for the treatment of currently intractable diseases and to maintain intestinal health. Furthermore, as exposed miRNAs are easy to degrade during long-distance transportation, EVs are a common and dependable carrier. The application of EVs and miRNAs in disease treatment involves two aspects. First, since EVs can be secreted by all cells, they may serve as a diagnostic marker for diseases. Specific miRNAs in EVs have diagnostic or prognostic potential for many diseases [69]. Second, EVs can deliver cargo to recipient cells over long distances, and marker proteins, such as CD63, on EVs facilitate their immune capture and enrichment; thus, many studies have reported their application in disease treatment [69,70]. EVs loaded with miRNAs have been used to treat various diseases in many animal models [71]. In the following discussion, we will focus on the application of EVs and their encapsulated miRNAs in the treatment of major intestinal diseases (Figure 3).

### 5.1. Inflammatory Bowel Diseases

The gastrointestinal tract is the chief immune organ of paramount importance for host health. Inflammation is a lethal threat to intestinal health and a critical part of the complex biological response when stimuli attack the intestine. Inflammation refers to a crucial consequence of the immune response when the host is attacked by pathogens; however, this process needs to be controlled in the case of oversecreted cytokines, which are detrimental to gut health and the immune system [72]. Moreover, inflammation is the cause of many diseases, such as necrotizing enterocolitis and diarrhea [73]. IBD is a common chronic gastrointestinal disease that mainly comprises ulcerative colitis (UC) and Crohn’s disease (CD). Germline genetics, the immune system, environmental factors, and intestinal microbiota are closely associated with IBD pathogenesis [74]. Among these factors, intestinal microbiota has recently received increasing attention. Patients with UC and CD have different intestinal microbiota compositions compared to that of healthy individuals. The abundance of *Leuconostocaceae* in CD and *Ruminococcaceae* in UC, which can produce SCFA, are decreased, whereas pro-inflammatory microbiota, such as *Enterobacteriaceae* in CD, is increased [75]. Therefore, adjusting the interaction between microbiota and intestine may be pivotal for the pathogenesis of IBD and could be a breakthrough point to cure IBD.

Recently, the role of EVs and miRNAs in the regulation of microbiota homeostasis and the development of intestinal inflammation was reported. EVs derived from host IEC, immune cells, and microbiota are both critical mediators in IBD, which would exert an immense role in the pathogeny and therapeutics of IBD. Exogenous miRNA transplantation can play a non-trivial role in shaping gut microbes. Ginger-derived miRNAs can promote more *L. rhamnosus* colonization in the intestinal lumen rather than mucosa, which is beneficial to restore colitis, thus promoting intestinal barrier function [32,76]. Dicer-specific knockout mice without the expression of miRNA-processing enzymes, exhibit exacerbated colitis. However, fecal miRNA presented within EV transplantation in wild-type mice restores the impaired intestinal flora and rescues colitis [53]. Specifically, oral administration of fecal miR-142-3p can increase the abundance of *L. reuteri* in the gut, which is beneficial for the recovery of colitis [58]. MiRNAs, separate or wrapped in EVs, such as miR-31, miR-215, miR-22, and miR-19b, can be used as biomarkers for IBD [77]. Specifically, some miRNAs have anti-inflammatory effects, and restoring their abundance is beneficial for attenuating inflammation. The expression of *miR-10b* in the intestinal villus upper cells and crypt cells decreased in a post-weaning piglet model as the intestinal inflammation intensified. Moreover, miR-10b knockout mice were more susceptible to DSS, indicating the anti-inflammatory potential of miR-10b [78]. MiR-143 and miR-145 couple with cAMP-responsive element-binding protein H (CREBH), which expression can be enhanced by *A. muciniphila*, to alleviate intestinal inflammation [61]. Researchers have also investigated the effects of exosomal miRNAs in breast milk on intestinal inflammation. They found that exosomal miR-4334 and miR-219 reduced intestinal inflammation, and miR-338 inhibited cell apoptosis induced by LPS. When co-transfected with these miRNAs, synergistic effects were observed in the prevention of apoptosis [79]. Notably, overexpression of certain miRNAs may aggravate inflammation. For example, a miR-30d inhibitor can attenuate inflammatory injury in IPEC-J2 cells caused by *Clostridium perfringens* beta2 (CPB2) toxin, which can cause necrotizing enterocolitis [73]. The regulatory function of EVs and miRNAs in IBD may occur through the induction of macrophage differentiation into the M1 pro-inflammatory phenotype, as indicated by EVs derived from *Fusobacterium nucleatum* [80].

The communication between miRNAs, EVs, and microbes affects the etiology of IBD. Probiotics can alleviate inflammation by regulating miRNA expression [81], whereas pathogenic bacteria disturb the miRNA expression profile and induce inflammation [62]. The effects of miRNAs on IBD are usually realized by regulating the intestinal immune system. The aryl hydrocarbon receptor (AhR) ligand is a product of tryptophan metabolism by intestinal microorganisms. AhR is an important component of intestinal immunity, and AhR ligands regulate T cell differentiation, which can be mediated by miRNA-132 [82]. MiR-31 can bind to 3′-UTRs of the genes encoding receptors for both IL-7 and IL-17 to relieve intestinal inflammation, and its expression is increased in colon tissues of patients with CD and UC [83]. miR-30c and miR-130a are targets of adherent-invasive *E. coli*, which prevents IEC autophagy by downregulating these two miRNAs and improving the survival of inflammatory cells [84]. The intestinal barrier is an essential part of the innate immune system, and a defective intestinal barrier is one of the pathogeneses of IBD [85]. Commensal bacteria can upregulate the expression of miR-21-5p, which increases the intestinal permeability by targeting ADP ribosylation factor 4 [86]. *F. nucleatum*-infected IEC-derived EVs delivered miR-129-2-3p to uninfected IEC, resulting in intestinal barrier dysfunction and inflammation [87]. Furthermore, pathogenic bacteria can inhibit autophagy-related gene expression in inflammatory cells by regulating the levels of miRNAs that promote the replication of inflammatory cells [88]. These studies suggest that unraveling the interaction mechanisms between miRNAs and gut microbes could provide new insights into the role of exosomal miRNAs in the prevention and treatment of intestinal inflammation.

### 5.2. Diarrhea

Diarrhea is a common clinical manifestation of various diseases caused by several factors, such as *Cryptosporidium*, fungi, bacteria, epidemic diarrhea virus (PEDV), diet, and genetic mutations [89,90,91,92,93]. Diarrhea can be chronic or acute, resulting in a series of adverse symptoms, including septicemia, peritonitis, electrolyte disorders, hypoalbuminemia, and anemia. EVs and their encapsulated miRNAs play critical roles in the diagnosis and treatment of diarrhea.

Diarrhea-predominant irritable bowel syndrome (IBS-D) is a chronic functional gastrointestinal disorder caused by genetic susceptibility, psychological factors, visceral hypersensitivity, increased mucosal permeability, and abnormal gut microbiology [94]. New insights into the pathogenesis of IBS-D suggest that EVs and miRNA-mediated signaling is essential. MiR-29a expression increased, while the tight junction proteins, ZO-1 and CLDN1, decreased in both in vivo and in vitro inflammatory models, indicating that miR-29a is involved in the pathogenesis of IBS-D by regulating intestinal barrier function [95]. MiR-16 can relieve IBS-D by inhibiting its target TLR4/NF-κB pathway [96]. In an IBS-D mouse model, miR-200a downregulates the expression of cannabinoid receptor 1 and serotonin transporters, thereby resulting in visceral hypersensitivity [97]. *C. perfringens* type C and *E. coli F18* are widely studied pathogenic bacteria that cause severe diarrhea. Certain miRNAs, including miR-532-3p, miR-218-3p, and miR-500, are associated with diarrhea caused by these pathogens. These miRNAs participate in pathways related to diarrhea and regulate the expression of essential proteins involved [98,99,100]. For instance, miR-532-3p and miR-133 increase the susceptibility of *C. perfringens* type C by directly targeting *NFATC4*, a critical gene in the Wnt signaling pathway [100]. Additionally, long non-coding RNA (lncRNA) interact with miRNAs involved in the pathogenesis of diarrhea. *MiR-122-5p* is a diarrhea-related gene, and its overexpression aggravates *C. perfringens* type C-induced diarrheal injury. The ALDB-898 lncRNA completely bind to miR-122–5p and thereby mitigate diarrheal injury [101]. This suggests that miRNAs are promising therapeutic targets in IBS-D.

Diarrheal diseases pose a significant threat to livestock and cause serious economic loss [102]. EVs isolated from Vero cells infected with PEDV showed different miRNA expression levels compared to those of the control cells. Researchers have found that 80 miRNAs are upregulated and 35 miRNAs are downregulated in the EVs of infected cells [92], indicating that miRNAs are critically involved in diarrheal diseases and could be potential biomarkers for diarrhea prediction. MiRNAs can interact with the gut microbiota to regulate the occurrence of diarrhea. Host-sourced miR-425-5p and miR-423-3p could target the fumarate reductase (*frd*) gene in the *Prevotella* genus, and their reduction in the colons of piglets resulted in the overaccumulation of succinate by the *Prevotella* genus, further causing diarrhea [57]. Notably, BEVs alleviate diarrhea by modulating macrophage function. *L. johnsonii*-derived EVs ameliorate diarrheal symptoms by enhancing M2 macrophage polarization and promoting gut barrier homeostasis [48]. *L. mucosae*-derived EVs can also alleviate piglet diarrhea in a macrophage phenotype-dependent manner [49]. Exosomal ssc-miR-1343 inhibits FAM131C expression, thereby suppressing the innate immune response and reducing PEVD replication [103]. However, whether the miRNAs encapsulated in these EVs critically mediate their beneficial effects in diarrhea requires further elucidation.

Due to the close correlation between miRNA and diarrhea, experimental studies have been conducted to investigate the advantages of vector-delivered artificial miRNA against PEDV. The results showed that the transient expression of two artificial miRNAs, *miR-349* and *miR-1447*, markedly decreased the expression of PEDV RNA and protein in African green monkey kidney cells [104]. Overall, vector-delivered miRNAs represent a novel therapeutic strategy for the treatment of diarrhea.

### 5.3. Colorectal Cancer

Colorectal cancer (CRC) is a common malignant tumor of the digestive tract with the second-highest mortality rate among cancers. Recently, as the incidence rate of CRC has increased [105], a variety of therapeutic approaches, such as surgery, radiation, and chemotherapy, have been applied in clinical practice. Phenotypic symptoms in the early stages of CRC are difficult to detect; therefore, EVs and their encapsulated miRNAs have been extensively researched as potential diagnostic and therapeutic biomarkers to promptly understand CRC progression. EVs play an important role in shaping the tumor microenvironment and act as critical participants in intercellular crosstalk by transmitting miRNAs from cancer or stromal cells to recipient cells to regulate the expression of oncogenes and tumor suppressor genes [106]. Several exosomal miRNAs, including miR-19a, miR-149-3p, miR-607-5, and miR-1246, are aberrantly expressed in patients with CRC compared to healthy individuals and those with other gastrointestinal diseases, implying the potential of miRNAs as biomarkers [107,108]. MiRNA dysregulation is related to survival after rectal cancer diagnosis. MiR-1 and miR-101-3p expression are associated with poor survival in patients with rectal cancer, indicating that the presence of miR-1 and miR-101-3p can be the basis for evaluating the prognosis of CRC [109]. MiRNAs regulate the pathogenesis and survival of CRC primarily by influencing the canonical pathway and tumor immune microenvironment [110]. MiR-34a binds to IL-6R, which ligand activates the oncogenic STAT3 transcription factor to relieve CRC progression [111]. Another miR-34 family member, *miR-34a-5p*, acts as a tumor suppressor by regulating DNA methylation [112]. MiR-342-5p could target placental growth factor (PGF), which participates in the activation of the mitogen-activated protein kinase (MAPK) pathway that regulates carcinogenesis, tumor cell invasion, and metastasis [113,114]. MiR-342-5p can reduce PGF expression, thus inhibiting CRC cell migration and invasion [115]. Additionally, the protein kinase B (AKT)/mammalian target of rapamycin (mTOR) pathway and Wnt/β-catenin pathway, which are both involved in the carcinogenic process of CRC, are pivotal targets of many miRNAs [116,117,118]. Furthermore, miRNAs play crucial roles in the host–intestinal microbiota crosstalk in CRC. *F. prausnitzii* promoted apoptosis and diminished the proliferation of CRC cells by producing butyrate. Butyrate can inhibit *miR-92a* transcription, thus increasing the p57 levels to suppress tumor activity [119]. Fecal microbiota transplantation (FMT) is a potential strategy to modulate the intestinal microbiome and regulate the immune system, making it a promising approach for CRC management [120]. As research on FMT progresses, it has been discovered that the mechanism also involves the transfer of miRNAs, as patients with CRC exhibit distinct fecal miRNA profiles compared to healthy individuals [107]. Mechanistically, fecal miRNAs influence the intestinal microbiota through two primary mechanisms. First, they can alter the composition and function of the microbiota, which, in turn, affects cytokine secretion. Second, they directly regulate the expression of components of the intestinal barrier, thereby influencing the microbiota [121]. Specifically, fecal miR-515-5p and miR-1226-5p can enter *Fusobacterium nucleatum* and *Escherichia coli*, promoting bacterial proliferation by regulating gene expression, which consequently exacerbates CRC [53,122,123]. These findings suggest that miRNAs can directly alter the expression of tumor-related genes and reshape the intestinal microbiota to interfere with CRC. However, our knowledge of the vast number of miRNAs and intestinal microbiota associated with CRC remains limited.

## 6. Conclusions

The exosomal miRNA–microbiota–host network is important for a variety of physiological processes. The microbiota can directly or indirectly influence intestinal health, and its dysregulation can be attributed to various intestinal diseases. As a pivotal mediator, exosomal miRNAs act as a bridge for communication between the gut and microorganisms. Additionally, EVs and encapsulated miRNAs are critical in gastrointestinal disorders, mediating crosstalk between the host and microbiota.

## 7. Perspective

Microorganisms and miRNAs are highly sensitive to alterations in each other’s abundance and are closely related to the pathology of intestinal diseases. Additionally, miRNAs are promising materials for designing disease-specific molecular signatures to achieve non-invasive screening tests because of their high sensitivity, specificity, and easy accessibility, which are of great help in the diagnosis, prognosis, and therapy of diseases. Due to the accuracy of nucleic acid pairing, miRNAs can precisely regulate the specific gene expression of microbes and their metabolism. Alterations in miRNA abundance can reflect disease progression in real time, enabling the adjustment of treatment strategies over time. Therefore, miRNAs and microbiota are increasingly valued as diagnostic and therapeutic tools for diseases.

However, there are still many practical issues regarding the application of miRNAs and microbiota in diseases that need to be addressed. First, we only discussed three diseases related to the miRNA–microbiota–host network. However, owing to the large population of both miRNAs and microorganisms, it is necessary to investigate the relationship between miRNAs, microbiota, and various diseases in populations of different age groups, countries, and dietary habits. Furthermore, the complex underlying mechanisms of miRNAs within cells and their roles in various diseases have yet to be fully elucidated. Second, biosensors may provide novel alternatives for miRNA analysis because of their high sensitivity. However, many problems still need to be solved, including their high cost, no portability, and high signal-to-noise ratio. Finally, some plant miRNAs have been identified as effective in treating animal gastrointestinal diseases. Therefore, whether miRNAs can achieve species cross-communication is a future research hotspot. Moreover, a multifunctional and accurate delivery system is required to ensure that exosomal miRNAs are delivered to target cells and microorganisms. The sophisticated sorting mechanisms of exosomal miRNAs are not comprehensively understood. The relationship between miRNAs and microorganisms is not one to one; therefore, how to accurately deliver miRNAs to microorganisms and regulate the abundance of target microorganisms remains to be explored. It is difficult to regulate specific miRNA expression in the target intestinal segment and precisely guide endogenous and exogenous miRNAs to intestinal lesions. Further studies are expected to improve EV–miRNA-based targeted therapies. These challenges have hindered the clinical application of EVs and miRNAs. The full potential of EVs and encapsulated miRNAs still requires further exploration.

## Figures and Tables

**Table 1:** The mechanisms of BEVs mediating host–microbiota crosstalk.

Microbiota	Encapsulated Substances	Role	References
*Lactobacillus plantarum*	sRNA71	Modulate cellular protein expression	[40]
*Bacteroides thetaiotaomicron*	protein	Target various immune cells	[44]
*E. coli Nissle 1917*, *Fusobacterium nucleatum*	peptidoglycans, outer membrane protein	Activate NF-κB pathway	[46]
*Limosilactobacillus mucosae*, *Lactobacillus johnsonii*	*/*	Modulate the macrophage and intestinal epithelial barrier	[48,49]
*Bifidobacterium longum*	protein	Induce anti-inflammatory cytokines secretion	[51]

**Table 2:** The mechanism of EVs and encapsulated miRNAs mediating host–microbiota crosstalk.

EVs and miRNAs	Microbiota	Role	References
Ginger-derived EVs	*Lactobacillaceae*, *Bacteroidales*, *Clostridiaceae*	Improve intestinal barrier function	[32]
miR-130a, miR-30c	*E. coli*	Inhibit autophagy-related proteins	[34]
miRNA-142a-3p	*Lactobacillus reuteri*	Regulating expression of DNA polymerase I and primase	[58]
miR-515-5p	*Fusobacterium nucleatum*	Directly increase microbiota 16S rRNA/23S rRNA transcripts	[53]
miR-159 and miR-143	*Lactobacillus fermentum*	Preserving the intestinal barrier function	[59]
miR-143 and miR-145	*Akkermansia muciniphila*	Promote IEC regeneration and barrier integrity	[61]
miR-214, miR-331-3p, miRNA-125a	*Salmonella enterica*	Induce immune response	[63,64]

## Data Availability

No new data were created or analyzed in this study. Data sharing is not applicable to this article.

## Abbreviations

EVs, extracellular vesicles; miRNAs, microRNAs; IBD, inflammatory bowel disease; CREBH, cAMP-responsive element-binding protein H; CRC, colorectal cancer; SCFAs, short-chain fatty acids; LPS, lipopolysaccharides; TLR4, toll-like receptor 4; CD, cluster of differentiation; PGLYRP, peptidoglycan recognition proteins; IECs, intestinal epithelial cells; QS, Quorum sensing; AI-2, autoinducer 2; MVBs, multivesicular bodies; IPEC-J2, porcine epithelial cell lines; BEVs, bacteria can also secrete bacterial extracellular vesicles; sRNA, small RNA; UTR, untranslated region; RISC, miRNA-induced silencing complex; NLRP3, NLR family pyrin domain-containing 3; DSS, dextran sulfate sodium; WT, wild-type; MyD88, myeloid differentiation primary response gene 88; UC, ulcerative colitis; CD, Crohn’s disease; CPB2, Clostridium perfringens beta2; AhR, aryl hydrocarbon receptor; PEDV, epidemic diarrhea virus; IBS-D, diarrhea-predominant irritable bowel syndrome; lncRNA, long non-coding RNA; frd, fumarate reductase; PGF, placental growth factor; MAPK, mitogen-activated protein kinase; AKT, protein kinase B; mTOR, mammalian target of rapamycin.
